# Association of amino acids related to urea cycle with risk of diabetic nephropathy in two independent cross-sectional studies of Chinese adults

**DOI:** 10.3389/fendo.2022.983747

**Published:** 2022-09-08

**Authors:** Penglong Cao, Bing Huang, Mo Hong, Yuxin Jiang, Ran Cao, Chen Chi, Yunfeng Cao, Shijun Li

**Affiliations:** ^1^ Clinical Laboratory, The First Hospital of Dalian Medical University, Dalian, China; ^2^ Research Department, Dalian Innovation Center of Laboratory Medicine Mass Spectrometry Technology, Dalian, China; ^3^ Research Department, Clinical Mass Spectrometry Profession Technology Innovation Center of Liaoning Province, Jinzhou, China; ^4^ Dalian Innovation Center of Laboratory Medicine Mass Spectrometry Technology, Dalian, China; ^5^ Shanghai Institute of Planned Parenthood Research, Shanghai, China; ^6^ Dalian Institute of Chemical Physics, Chinese Academy of Sciences, Dalian, China

**Keywords:** diabetic nephropathy, urea cycle, amino acids, association, cross-sectional studies

## Abstract

**Objective:**

To investigate the association between amino acids related to the urea cycle and diabetic nephropathy (DN) in two independent cross-sectional studies.

**Methods:**

We obtained the medical records of 145 individuals with DN and 596 individuals without DN who attended an annual health examination at Liaoning Medical University First Affiliated Hospital (LMUFAH), China, from May 2015 to August 2016. From April 2018 to April 2019, we collected medical records of another 741 individuals: 338 individuals with DN and 403 individuals without DN from the Second Affiliated Hospital of Dalian Medical University (DALIAN), China. Binary logistic regression was used to obtain the odds ratio (OR) and 95% confidence interval (CI).

**Results:**

In two independent cross-sectional studies, we observed that citrulline was consistently associated with DN risk [OR (95% CI) of per standard deviation (SD) increase for citrulline in the LMUFAH population: 1.200 (1.006, 1.432); OR (95% CI) of per SD increase for citrulline in the DALIAN population: 1.189 (1.012, 1.396); pooled effect size for citrulline: 1.194 (1.060, 1.345)]. However, ornithine, arginine, and the ratio of arginine to ornithine were consistently unrelated to DN risk, and the ratios of other amino acids in the urea cycle were inconsistently associated with DN risk.

**Conclusions:**

Citrulline was consistently associated with DN risk in two independent cross-sectional studies in Chinese adults.

## Introduction

When the pancreas does not produce enough insulin or the body is unable to use insulin effectively, symptoms such as excessive urine excretion (polyuria), thirst (polydipsia), frequent hunger pangs, weight loss, loss of vision, and fatigue may occur; we call this disease diabetes (1–3). Diabetes can be classified according to its cause into type 1 diabetes (insufficient insulin production, common in children and adolescents) and type 2 diabetes (T2D; insulin resistance, common in obese adults), with over 95% of people with diabetes experiencing T2D. In recent years, diabetes has become a severe, worldwide public health problem: in 2019, diabetes was the ninth leading cause of death worldwide, directly causing an estimated 1.5 million deaths, and 48% of diabetes deaths occur before the age of 70 years (4). This means that diabetes-related research is imperative and significant.

Diabetic nephropathy (DN) is a result of a complex interaction between metabolic processes, is a result of long-term tubular (5, 6) and glomerular damage, and is an important cause of renal failure (7) and inflammatory and hemodynamic change (3). Its key risk factor is insulin resistance.

Early studies have found that 5%–40% of T2D patients eventually develop DN (8), which is the leading cause of end-stage renal disease (ESRD) worldwide (9, 10). The increased risk of cardiovascular death in patients with diabetes is mainly associated with the presence of DN (11). In addition, research has shown that DN has become the main cause of chronic kidney disease in China (12), and it is important to note that once the disease is established it can only progress; it cannot be cured (13). All in all, DN poses a major burden on healthcare systems and the global economy (14).

It is well known that disease leads to changes in the pathophysiological processes of the body, which ultimately cause corresponding changes in metabolites. Furthermore, metabolites reflect the environment in which the cells live, which in turn is closely related to the nutritional status of the cells, the effects of drugs and environmental pollutants, and the influence of other external factors. Therefore, analyzing certain metabolites and comparing them with those of non-diseased individuals to find biomarkers of the disease will provide a better approach to disease diagnosis.

Considering the seriousness of the dangers of DN, we urgently need to find biomarkers that can identify populations at high risk of DN and predict this disease at an early stage. In recent decades many novel biomarkers related to diabetes and its complications were found through metabolomics (1). Experimental evidence developed in murine models and cell culture suggests that urea reduces insulin sensitivity and suppresses insulin secretion (15). Moreover, a recent study showed that higher levels of blood urea nitrogen were associated with an increased risk of developing diabetes mellitus (16). Arginine, citrulline, and ornithine belong to urinary metabolites and substrates. Some studies showed that amino acids related to the urea cycle were associated with inflammatory markers and oxidative stress (17, 18). Our previous study found that amino acids related to the urea cycle were associated with T2D in Chinese adults (19). However, whether amino acids related to the urea cycle are associated with the risk of incident DN remains unclear.

In this study, we aimed to explore whether plasma levels of amino acids related to the urea cycle were associated with DN risk using two independent cross-sectional surveys.

## Material and methods

### Subjects

Liaoning Medical University First Affiliated Hospital (LMUFAH) is a national comprehensive tertiary class A hospital in China. By retrieving electronic medical records, data from 741 T2D patients (596 non-DN and 145 DN patients) were collected from May 2015 to August 2016. The Second Affiliated Hospital of Dalian Medical University (DALIAN) is the largest comprehensive grade 3A hospital in the Southern Liaoning province of China, with nearly 2 million emergency department visits per year. In accordance with the inclusion and exclusion criteria below, we collected data from 741 T2D patients (403 non-DN and 338 DN patients) to address our research questions. Diabetes patients were diagnosed in accordance with the World Health Organization’s criteria from 1998 (20).

**Figure 1:**
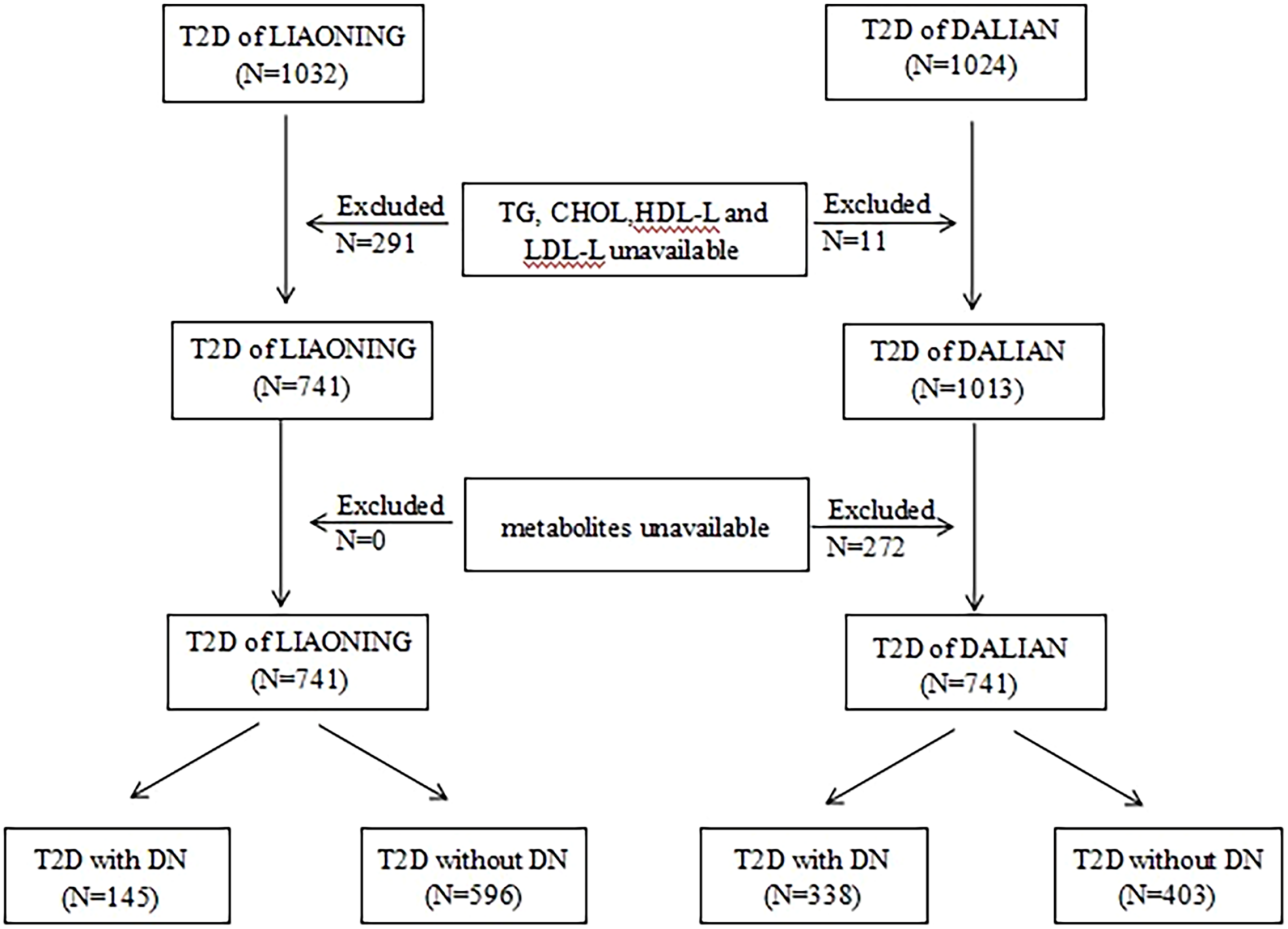
Flow chart of selection of participant in this study. T2D, type 2 diabetics; LMUFAH, Liaoning Medical University First Affiliated Hospital; DALIAN, Second Affiliated Hospital of Dalian Medical University; TG, triglyceride; CHOL, cholesterol; HDL-C, high-density lipoprotein cholesterol; LDL-C, low-density lipoprotein cholesterol; DN, diabetic nephropathy.

In both cross-sectional studies, the inclusion criteria were as follows: (1) for patients in the non-DN group, a diagnosis of T2D without DN, and (2) for patients in the DN group, a diagnosis of T2D with DN (21). The exclusion criteria were as follows: (1) a lack of information on triglyceride (TG), cholesterol (CHOL), high-density lipoprotein cholesterol (HDL-C), and low-density lipoprotein cholesterol (LDL-C) levels; (2) a lack of information on metabolites; and (3) age under 18 years. The specific process is shown in **Figure 1**.

We obtained the data from the hospitals’ computer systems, including demographic characteristics, anthropometric measurements, relevant clinical measurements, and metabolism measurements. Demographic characteristics data included age, gender, current smoking status, and current drinking status. Anthropometric measurements included weight, height, and blood pressure. Clinical measurements on lipid profiles were collected. Metabolism measurements included amino acids related to the urea cycle (i.e., ornithine, arginine, citrulline). Anti-diabetic measures included oral anti-diabetic agents and insulin. The statuses “current smoker” and “current drinker” indicate patients who currently smoke and currently drink alcohol, respectively, regardless of whether they have reduced their consumption. There were no major changes in treatment and mainstream medications for people with diabetes, with negligible differences over time.

The protocol of the study was approved by the Ethics Committee for Clinical Research of the LMUFAH and the Ethics Committee for Clinical Research of the DALIAN. Informed consent was waived because of the nature of the retrospective study, which was in accordance with the Helsinki Declaration of 1964 and its later amendments. 

### Data collection

Height, weight, and blood pressure were measured by specially trained doctors and nurses using standardized methods. Patients had to remove heavy clothing and shoes while weight and height measurements were obtained. Weight and height measurements were accurate to one decimal point. Blood pressure was not measured until patients had been resting in a sitting position for at least 10 minutes. Body mass index (BMI) was calculated by dividing weight in kilograms by squared height in meters.

After a continuous fasting period of at least 8 hours, patients’ blood samples were collected. Lipid profiles were assayed by an automatic biochemical analyzer, including TG, CHOL, HDL-C, and LDL-C. Metabolomic profiles of amino acids including ornithine, arginine, and citrulline were assayed.

### Measurement of plasma amino acids related to urea cycle

Methods for the measurement of plasma amino acids have already been discussed in detail in previous publications (22). Briefly, each patient had capillary whole blood drawn after at least 8 hours of fasting; the sample was stored as a dried blood spot and used in the assay of metabolomics. Metabolites in a dried blood spot were measured by direct infusion using mass spectrometry technology and the AB Sciex 4000 QTrap System (AB Sciex, Framingham, MA, USA). We used high-purity water and acetonitrile from Thermo Fisher Scientific (Waltham, MA, USA) as a diluting agent and mobile phase. Samples were 1-butanol and acetyl chloride from Sigma-Aldrich (St Louis, MO, USA). Isotope-labeled internal standard samples of 12 amino acids (NSK-A) were purchased from Cambridge Isotope Laboratories (Tewksbury, MA, USA) and standard samples of the amino acids were purchased from Chromsystems Instruments & Chemicals GmbH (Gräfelfing, Germany).

### Statistical analysis

Continuous data of normal distribution were expressed as mean ± standard deviation (SD); if normal distributions were not accepted, the data were expressed as the median (interquartile range). Qualitative data were presented as a number (percentile). Mann–Whitney *U*-tests or Student’s *t*-tests were used to compare differences in continuous data. Chi-squared tests were used to compare the differences in qualitative data. Binary logistic regression was used to obtain odds ratios (ORs) and their 95% confidence intervals (CIs). A structured adjustment scheme was used to control for confounders: model 1, unadjusted; model 2, adjusted for gender and age; model 3, adjusted for covariates in model 2 plus BMI and systolic blood pressure (SBP); model 4, adjusted for covariates in model 3 plus TG, CHOL, HDL-C, and LDL-C; and model 5, adjusted for covariates in model 4 plus anti-diabetic measures. Metabolites’ associations with DN risk were pooled with an inverse variance-weighted fixed-effect meta-analysis. Before being introduced into regression models, amino acids related to the urea cycle and their ratios were scaled to SD concentrations. All statistical analyses were performed using the Statistical Analysis System 9.4 (SAS Institute Inc., Cary, NC, USA). A two-tailed p-value <.05 was considered statistically significant.

## Results

### Characteristics of the population

The clinical characteristics and the baseline demographics of the study individuals from the two populations are presented in **Table 1**. The LMUFAH patients had a median age of 59.00 years (interquartile range: 50.00 to 68.00 years) and a median BMI of 25.10 kg/m² (interquartile range: 22.85 to 27.46 kg/m²). Levels of SBP, HDL-C, anti-diabetic measures, citrulline, and citrulline/ornithine for those in the DN group were significantly higher than for those in the non-DN group. The arginine–citrulline ratio for those in the DN group was significantly lower than for those in the non-DN group. However, there was no significant difference in gender, age, current smokers, current drinkers, BMI, diastolic blood pressure (DBP), TG, CHOL, LDL-C, ornithine, arginine, or arginine–ornithine ratio between the DN and non-DN groups.

**Table 1:** Clinical and biological characteristics of all patients.

	LMUFAH (N = 741)			DALIAN (N = 741)		
	Non-DN (N = 596)	DN (N = 145)	p-value	Non-DN (N = 403)	DN (N = 338)	p-value
Gender (male)	319 (53.52%)	72 (49.66%)	0.403	218 (54.09%)	167 (49.41%)	0.204
Age (years)	58.5 (50.0, 67.0)	59.0 (50.0, 69.0)	0.359	60.0 (51.0, 66.0)	63.0 (53.0, 70.0)	0.000
Current smoker, n (%)	193 (32.38%)	44 (30.34%)	0.637	78 (19.35%)	57 (16.86%)	0.382
Current drinker, n (%)	164 (27.52%)	41 (28.28%)	0.855	41 (10.17%)	31 (9.17%)	0.646
BMI (kg/m²)	25.0 (22.7, 27.4)	25.7 (22.9, 27.7)	0.316	25.7 (23.9, 29.0)	26.6 (24.4, 29.2)	0.039
SBP (mmHg)	138.0 (122.0, 154.0)	142.0 (126.0, 162.0)	0.010	144.0 (131.0, 154.0)	151.0 (136.0, 169.0)	0.000
DBP (mmHg)	81.0 (74.0, 90.0)	82.0 (73.0, 91.0)	0.710	81.0 (73.0, 89.0)	80.0 (74.0, 89.0)	0.888
TG (mmol/l)	1.64 (1.10, 2.38)	1.72 (1.21, 2.39)	0.396	1.56 (1.04, 2.10)	1.61 (1.15, 2.74)	0.050
CHOL (mmol/l)	4.62 (3.84, 5.27)	4.80 (3.92, 5.57)	0.077	4.99 (4.24, 5.67)	4.92 (4.06, 5.88)	0.420
HDL-C(mmol/l)	1.00 (0.84, 1.24)	1.06 (0.90, 1.30)	0.018	1.18 (1.00, 1.39)	1.14 (0.95, 1.33)	0.046
LDL-C(mmol/l)	2.78 (2.19, 3.37)	2.79 (2.27, 3.49)	0.205	2.58+0.81	2.48+0.88	0.105
Anti-diabetic measures	538 (90.27%)	144 (99.31%)	0.000	320 (79.40%)	302 (89.35%)	0.000
Orn, µmol/l	17.63 (13.18, 23.73)	17.45 (12.63, 23.41)	0.820	11.31 (8.56, 14.92)	11.29 (8.28, 14.46)	0.716
Arg, µmol/l	9.77 (5.43, 17.17)	10.08 (5.70, 15.98)	0.628	2.79 (1.75, 4.34)	3.11 (1.78, 4.42)	0.113
Cit, µmol/l	19.56 (15.20, 25.33)	22.03 (16.98, 27.50)	0.002	22.57 (17.45, 27.69)	22.78 (17.00, 30.58)	0.194
Arg/Orn	0.57 (0.28, 0.98)	0.57 (0.29, 0.90)	0.818	0.24 (0.15, 0.37)	0.26 (0.18, 039)	0.038
Cit/Orn	1.10 (0.77, 1.50)	1.25 (0.94, 1.69)	0.002	2.03 (1.45, 2.68)	2.10 (1.54, 2.83)	0.051
Arg/Cit	0.53 (0.30, 0.85)	0.44 (0.26, 0.70)	0.010	0.13 (0.08, 0.18)	0.13 (0.08, 0.20)	0.587

Orn, ornithine; Arg, arginine; Cit, citrulline; Arg/Orn, arginine–ornithine ratio; Cit/Orn, citrulline–ornithine ratio; Arg/Cit, arginine–citrulline ratio.

Data are mean (standard deviation), median (interquartile range) to n (%).

p-values <.05 were considered statistically significant.

The DALIAN patients had a median age of 61.00 years (interquartile range: 53.00 to 68.00 years) and a median BMI of 26.22 kg/m² (interquartile range: 24.10 to 29.06 kg/m²). HDL-C levels for those in the DN group were significantly lower than for those in the non-DN group. Age, BMI, SBP, and anti-diabetic measures for those in the DN group were significantly higher than for those in the non-DN group. Moreover, gender; current smoking status; current drinking status; levels of DBP, TG, CHOL, LDL-C, ornithine, arginine, and citrulline; and the ratios of arginine to ornithine, citrulline to ornithine, and arginine to citrulline in both the DN and non-DN groups were not significantly different.

In the LMUFAH population and DALIAN population, SBP and anti-diabetic measures for those in the DN groups were significantly higher than for those in the non-DN groups, and HDL-C levels in both the DN and non-DN groups were significantly different.

### Associations of amino acids with diabetic nephropathy

In the LMUFAH population, citrulline was positively associated with DN risk in the univariate analyses. The association was still significant after further adjustment for traditional risk factors [OR (95% CI) of per SD increase for citrulline: 1.200 (1.006, 1.432)]. However, the associations of ornithine and arginine with DN risk were not significant.

In the DALIAN population, citrulline was positively associated with DN risk in the univariate analyses. After further adjustment for traditional risk factors, the association was still significant [OR (95% CI) of per SD increase for citrulline: 1.189 (1.012, 1.396)]. However, the associations of ornithine and arginine with DN risk were not significant.

In the fixed-effect pooled analysis, the associations of amino acids related to the urea cycle with DN risk were in accordance with findings in the LMUFAH and DALIAN populations. Citrulline was still positively associated with DN risk. The OR (95% CI) of per SD increase was 1.252 (1.121, 1.398) for citrulline in the univariate analyses. After further adjustment for traditional risk factors, the pooled effect size was 1.194 (1.060, 1.345) for citrulline. The associations of ornithine and arginine with DN risk were not significant (**Table 2**).

**Table 2:** The difference of amino acids related to the urea cycle by logistic regression in two independent cross-sectional studies.

	Univariate		Multivariate							
	Model 1		Model 2		Model 3		Model 4		Model 5	
	OR (95% CI)	p-value	OR (95% CI)	p-value	OR (95% CI)	p-value	OR (95% CI)	p-value	OR (95% CI)	p-value
LMUFAH, per 1 SD										
Orn, µmol/l	0.924 (0.757, 1.129)	0.441	0.925 (0.758, 1.130)	0.446	0.932 (0.760, 1.142)	0.496	0.943 (0.768, 1.157)	0.573	0.957 (0.774, 1.183)	0.682
Arg, µmol/l	0.896 (0.739, 1.085)	0.261	0.891 (0.732, 1.084)	0.249	0.862 (0.707, 1.051)	0.141	0.861 (0.705, 1.052)	0.144	0.853 (0.697, 1.043)	0.122
Cit, µmol/l	1.278 (1.082, 1.508)	0.004	1.261 (1.065, 1.493)	0.007	1.233 (1.039, 1.463)	0.017	1.202 (1.009, 1.432)	0.039	1.200 (1.006, 1.432)	0.042
DALIAN, per 1 SD										
Orn, µmol/l	0.960 (0.829, 1.111)	0.580	0.961 (0.829, 1.115)	0.603	0.964 (0.829, 1.121)	0.634	0.962 (0.826, 1.121)	0.619	0.953 (0.816, 1.114)	0.548
Arg, µmol/l	1.096 (0.948, 1.266)	0.215	1.092 (0.944, 1.264)	0.237	1.096 (0.944, 1.272)	0.227	1.099 (0.945, 1.279)	0.221	1.086 (0.933, 1.265)	0.285
Cit, µmol/l	1.232 (1.062, 1.428)	0.006	1.188 (1.019, 1.383)	0.027	1.162 (0.994, 1.359)	0.060	1.187 (1.012, 1.393)	0.035	1.189 (1.012, 1.396)	0.035
Pooled, per 1 SD										
Orn, µmol/l	0.947 (0.842, 1.066)	0.369	0.948 (0.842, 1.068)	0.380	0.953 (0.844, 1.075)	0.432	0.955 (0.845, 1.080)	0.463	0.954 (0.842, 1.082)	0.466
Arg, µmol/l	1.019 (0.908, 1.144)	0.751	1.016 (0.903, 1.142)	0.794	1.005 (0.892, 1.132)	0.936	1.006 (0.891, 1.135)	0.927	0.995 (0.881, 1.123)	0.933
Cit, µmol/l	1.252 (1.121, 1.398)	0.000	1.220 (1.090, 1.367)	0.001	1.194 (1.064, 1.340)	0.003	1.194 (1.061, 1.343)	0.003	1.194 (1.060, 1.345)	0.003

Orn, ornithine; Arg, arginine; Cit, citrulline.

Model 1, unadjusted.

Model 2, adjusted for gender and age.

Model 3, adjusted for covariates in model 2 plus BMI and SBP.

Model 4, adjusted for covariates in model 3 plus TG, CHOL, HDL-C, and LDL-C.

Model 5, adjusted for covariates in model 4 plus anti-diabetic measures.

### Associations between ratios of amino acids and diabetic nephropathy risk

The relationship between ratios of amino acids in the urea cycle and DN is presented in **Table 3**. In the LMUFAH population, the ratio of arginine to citrulline was significantly associated with DN risk in the univariate analyses and in the multivariate analyses [the OR (95% CI) of per SD increase for arginine–citrulline: 0.716 (0.571, 0.898)]. The ratio of citrulline to ornithine was associated with DN risk in the univariate analyses. After adjustment for confounders, the association was changed [the OR (95% CI) of per SD increase for citrulline–ornithine: 1.141 (0.950, 1.370)]. However, the association between the ratio of arginine to ornithine and DN risk was not significantly different.

**Table 3:** Ratios of amino acids related to the urea cycle for the risk of diabetic nephropathy.

	Univariate		Multivariate							
	Model 1		Model 2		Model 3		Model 4		Model 5	
	OR (95% CI)	p-value	OR (95% CI)	p-value	OR (95% CI)	p-value	OR (95% CI)	p-value	OR (95% CI)	p-value
LMUFAH, per 1 SD										
Arg/Orn,	0.918 (0.760, 1.109)	0.376	0.913 (0.753, 1.106)	0.354	0.884 (0.729, 1.072)	0.209	0.880 (0.724, 1.071)	0.202	0.876 (0.718, 1.068)	0.189
Cit/Orn	1.212 (1.023, 1.435)	0.026	1.191 (1.002, 1.415)	0.047	1.170 (0.983, 1.392)	0.077	1.137 (0.951, 1.361)	0.160	1.141 (0.950, 1.370)	0.158
Arg/Cit	0.732 (0.592, 0.905)	0.004	0.737 (0.594, 0.913)	0.005	0.714 (0.574, 0.887)	0.002	0.722 (0.579, 0.901)	0.004	0.716 (0.571, 0.898)	0.004
DALIAN, per 1 SD										
Arg/Orn	1.095 (0.947, 1.265)	0.221	1.094 (0.946, 1.266)	0.225	1.088 (0.939, 1.262)	0.262	1.090 (0.939, 1.266)	0.258	1.080 (0.930, 1.254)	0.316
Cit/Orn	1.213 (1.047, 1.405)	0.010	1.179 (1.016, 1.369)	0.030	1.147 (0.985, 1.334)	0.077	1.173 (1.006, 1.368)	0.042	1.192 (1.021, 1.392)	0.026
Arg/Cit	1.024 (0.886, 1.183)	0.746	1.051 (0.908, 1.217)	0.504	1.064 (0.917, 1.235)	0.412	1.054 (0.907, 1.225)	0.493	1.041 (0.894, 1.211)	0.607
Pooled, per 1 SD										
Arg/Orn	1.026 (0.915, 1.151)	0.663	1.024 (0.912, 1.150)	0.687	1.008 (0.896, 1.133)	0.901	1.007 (0.895, 1.134)	0.903	1.001 (0.889, 1.128)	0.984
Cit/Orn	1.213 (1.085, 1.355)	0.001	1.184 (1.058, 1.326)	0.003	1.157 (1.032, 1.297)	0.012	1.158 (1.030, 1.301)	0.014	1.170 (1.040, 1.317)	0.009
Arg/Cit	0.921 (0.817, 1.037)	0.175	0.939 (0.832, 1.060)	0.309	0.937 (0.829, 1.059)	0.299	0.935 (0.826, 1.059)	0.291	0.927 (0.817, 1.051)	0.238

Arg/Orn, arginine/ornithine; Cit/Orn, citrulline/ornithine; Arg/Cit, arginine/citrulline; Arg/Orn, arginine–ornithine ratio; Cit/Orn, citrulline–ornithine ratio; Arg/Cit, arginine–citrulline ratio.

Model 1, unadjusted.

Model 2, adjusted for gender and age.

Model 3, adjusted for covariates in model 2 plus BMI and SBP.

Model 4 adjusted for covariates in model 3 plus TG, CHOL, HDL-C, and LDL-C.

Model 5, adjusted for covariates in model 4 plus anti-diabetic measures.

In the DALIAN population, the association between the ratio of citrulline to ornithine and DN risk was significantly different in the univariate analyses and in the multivariate analyses [the OR (95% CI) of per SD increase for citrulline–ornithine: 1.192 (1.021, 1.392)]. However, the ratios of arginine to ornithine and arginine to citrulline were not associated with DN risk.

In the fixed-effect pooled analysis, the ratio of citrulline to ornithine was associated with DN risk in the univariate analyses and in the multivariate analyses [OR (95% CI) of per SD increase: 1.170 (1.040, 1.317)]. The associations between the ratios of other amino acids and DN risk were not significantly different.

## Discussion

One of the serious complications of T2D is DN; patients with DN have an increased risk of developing cardiovascular and cerebrovascular diseases (23, 24). However, discovering a single diagnostic marker of DN is still in the exploration stage. Thus, we urgently need biomarkers for the early diagnosis of DN. In this study, we tested associations between amino acids related to the urea cycle and DN risk. We observed that citrulline was consistently associated with DN risk in two independent cross-sectional studies.

A small study of 78 Japanese participants showed that the correlation coefficients for citrulline were significantly associated with the urinary albumin–creatinine ratio and estimated glomerular filtration rate (25). Consistent with this finding, we found that citrulline was associated with DN. Citrulline is efficiently taken up by the proximal renal tubules, where it is converted to urea *via* arginine (26). Urea is a major end product of nitrogen metabolism. Researchers have found a significant accumulation of urea cycle intermediates in patients with ESRD (27). Owing to the important role kidneys play in the conversion of citrulline to arginine, we speculate that increased serum levels of citrulline in individuals with DN could be related to the degradation of this function. Moreover, citrulline is involved in the synthesis of nitric oxide (NO) in the citrulline–NO cycle (26). NO is identified as an important regulator of renal function and morphology (28). Asymmetric dimethylarginine can be metabolized to citrulline, which potentially prevents the inhibition of endothelial nitric oxide synthase by asymmetric dimethylarginine (29). The elevation level of citrulline in DN patients may be related to dysregulation of the renal NO-producing system. Interestingly, the ratio of citrulline to ornithine was inconsistently associated with DN risk in two independent cross-sectional studies, whereas the pooled result was associated with DN risk. The findings need to be further explored in a broader population, and an analysis of serum NA levels will be helpful in figuring this out.

There are several limitations to this study. First, estimated glomerular filtration rates (eGFRs) have not been evaluated in the present study; therefore, direct evidence about the association between amino acids related to the urea cycle and DN is lacking. Second, the data we collected mainly focused on DN patients in Jinzhou and Dalian, which cannot necessarily be generalized to all DN patients in China. Third, our patients with T2D and DN were in-patients. Their condition might be more serious than that of general patients with T2D and DN. Finally, this study is cross-sectional design: experimental values of blood urea nitrogen, serum ammonia, serum creatinine, proteinuria, albuminuria, and dietary factors were not included in this study, so we cannot derive a causal relationship between amino acids related to the urea cycle and DN. In summary, the findings of the study need to be evaluated and validated in a broader population.

The present study has important implications for public health. DN is one of the major public health problems worldwide, and it has caused a huge burden on the global health system. Moreover, the incidence of progression of DN to ESRD is increasing (30). Therefore, it is very important to accurately predict the occurrence and development of DN. This study suggests that citrulline might be a candidate marker for future DN risk scoring if these findings can be replicated in cohort studies, especially in China.

In conclusion, citrulline was consistently associated with DN risk in two independent cross-sectional studies of amino acids related to the urea cycle and DN risk. As this was a cross-sectional study, it is necessary to confirm the findings through studies in other populations.

## Data availability statement

The raw data supporting the conclusions of this article will be made available by the authors, without undue reservation.

## Ethics statement

The studies involving human participants were reviewed and approved by the Ethics Committee for Clinical Research of the LMUFAH and the Ethics Committee for Clinical Research of the DALIAN. Written informed consent for participation was not required for this study in accordance with the national legislation and the institutional requirements.

## Author contributions

SL and YC conceived the project and designed the experiments. PC wrote the manuscript. BH analyzed data. MH, YJ, RC, and CC collected the information and contributed to the writing of this manuscript. All authors edited the final version of the manuscript. All authors contributed to the article and approved the submitted version.

## Funding

This work was supported by the National Key Research and Development Program of China (2019YFA0802302, 2019YFA0802300), the Special Fund of State Key Joint Laboratory of Environment Simulation and Pollution Control, the Development and application of clinical mass spectrometry detection technology for important small molecule metabolites (2020JH2/10300116), the Key R&D Program of Liaoning Province and the Dalian Laboratory Medicine Mass Spectrometry Technology Innovation Center. The funders had no role in study design, data collection and analysis, decision to publish, or preparation of the manuscript.

## Acknowledgments

The authors thank all doctors, nurses, and research staff at the Liaoning Medical University in Jinzhou and the Second Affiliated Hospital of Dalian Medical University for their participation in this study.

## Conflict of interest

The authors declare that the research was conducted in the absence of any commercial or financial relationships that could be construed as a potential conflict of interest.

## Publisher’s note

All claims expressed in this article are solely those of the authors and do not necessarily represent those of their affiliated organizations, or those of the publisher, the editors and the reviewers. Any product that may be evaluated in this article, or claim that may be made by its manufacturer, is not guaranteed or endorsed by the publisher.
